# APP Protein Family Signaling at the Synapse: Insights from Intracellular APP-Binding Proteins

**DOI:** 10.3389/fnmol.2017.00087

**Published:** 2017-03-30

**Authors:** Suzanne Guénette, Paul Strecker, Stefan Kins

**Affiliations:** ^1^Independent Researcher84 3099 Uplands Dr., Ottawa, ON, Canada; ^2^Department of Biology, Division of Human Biology, University of KaiserslauternKaiserslautern, Germany

**Keywords:** amyloid precursor protein (APP), FE65, FE65L1, X11/Mint proteins, synaptic signaling

## Abstract

Understanding the molecular mechanisms underlying amyloid precursor protein family (APP/APP-like proteins, APLP) function in the nervous system can be achieved by studying the APP/APLP interactome. In this review article, we focused on intracellular APP interacting proteins that bind the YENPTY internalization motif located in the last 15 amino acids of the C-terminal region. These proteins, which include X11/Munc-18-interacting proteins (Mints) and FE65/FE65Ls, represent APP cytosolic binding partners exhibiting different neuronal functions. A comparison of FE65 and APP family member mutant mice revealed a shared function for APP/FE65 protein family members in neurogenesis and neuronal positioning. Accumulating evidence also supports a role for membrane-associated APP/APLP proteins in synapse formation and function. Therefore, it is tempting to speculate that APP/APLP C-terminal interacting proteins transmit APP/APLP-dependent signals at the synapse. Herein, we compare our current knowledge of the synaptic phenotypes of APP/APLP mutant mice with those of mice lacking different APP/APLP interaction partners and discuss the possible downstream effects of APP-dependent FE65/FE65L or X11/Mint signaling on synaptic vesicle release, synaptic morphology and function. Given that the role of X11/Mint proteins at the synapse is well-established, we propose a model highlighting the role of FE65 protein family members for transduction of APP/APLP physiological function at the synapse.

## Introduction

The amyloid precursor protein (APP) can be processed to generate the amyloid β (Aβ) peptides, which aggregate to form senile plaques, one of the major pathological hallmarks found in Alzheimer’s disease (AD; Masters and Selkoe, 2012). APP is a ubiquitously expressed type I transmembrane protein with a large ectodomain, a single membrane spanning domain, and a short cytoplasmic tail. The ectodomain comprises two highly conserved E1 and E2 domains, involved in metal (copper and zinc) and heparin binding (Baumkötter et al., 2012; Müller and Zheng, 2012).

APP has important physiological functions at the synapse (Zheng and Koo, 2011). Aged mice deficient in APP show impairments in behavior (Müller et al., 1994; Phinney et al., 1999; Ring et al., 2007), long-term potentiation (LTP; Seabrook et al., 1999; Ring et al., 2007), dendritic branching and synaptic density (Zheng et al., 1995; Dawson et al., 1999; Phinney et al., 1999; Seabrook et al., 1999; Lee et al., 2010; Tyan et al., 2012; Weyer et al., 2014). No synaptic deficits are present in APP-like protein 2 (APLP2) knockout (KO) mice (Midthune et al., 2012; Weyer et al., 2014). Yet mice doubly deficient for APP and APLP2 or for APLP1 and APLP2 exhibit early postnatal lethality and show deficits in neuromuscular junction (NMJ) formation, including incorrect apposition of pre- and postsynaptic sites (von Koch et al., 1997; Heber et al., 2000; Wang et al., 2005; Klevanski et al., 2014). These data suggest genetic redundancy of APP family members for synapse formation.

Interestingly, the introduction of either sAPPα (APPsα-KI) or APP with a mutation in the intracellular domain (APPY682G) onto an APLP2-deficient background produced a partial rescue of the phenotypes presented in the doubly deficient mice (Barbagallo et al., 2011; Weyer et al., 2011). In addition, it was shown that learning deficits in *Drosophila* lacking APPL, the only homolog of APP in the fruit fly, are partially rescued by secreted sAPPL (788 amino acid soluble N-terminal fragment) or a non-cleavable full-length APPL (Bourdet et al., 2015; Cassar and Kretzschmar, 2016). These data indicate that APP function depends on both the activity of secreted sAPP, likely functioning as a ligand, and on full-length APP, possibly working as a receptor or co-receptor. Interestingly, exogenous Adenovirus-mediated expression of sAPPα in aged AD model transgenic mice (APPswe/PS1ΔE9) restored synaptic plasticity and partially rescued spine density deficits (Fol et al., 2016). These data, along with those from many other studies, suggest that sAPPα may function as a neurotrophic factor (Meziane et al., 1998; Bour et al., 2004; Taylor et al., 2008; Claasen et al., 2009; Weyer et al., 2014; Hick et al., 2015; Kundu et al., 2016; Plummer et al., 2016). Although many different extracellular binding partners of APP are reported, including different heparin sulfate proteoglycans (HSPG; Aydin et al., 2012; Reinhard et al., 2013), none of the identified proteins have been reported to function as sAPP receptors. In the case of full length APP, it was proposed that APP might be involved in trans-synaptic signaling, similar to other synaptic modulators such as Neuroligin, Neurexin and LRRTMs (Siddiqui and Craig, 2011; Baumkötter et al., 2012). Several studies provide experimental evidence consistent with this notion. Dimerization of APP can occur in a *trans*-orientation (Soba et al., 2005; Kaden et al., 2008; Wang Z. et al., 2009; Baumkötter et al., 2012; Klevanski et al., 2014) and inactivation of APP at either the pre- or postsynaptic sites of the NMJ in APLP2 KO mice causes defects similar to the combined germline deletions of APP and APLP2 (Wang Z. et al., 2009). Moreover, expression of APP bearing an intact E1 domain in human embryonic kidney cells co-cultured with primary hippocampal neurons promotes the presynaptic differentiation of contacting axons (Wang Z. et al., 2009; Baumkötter et al., 2014; Stahl et al., 2014). Dendritic spine formation is also increased by heterologous expression of APP in primary hippocampal neurons (Lee et al., 2010). Conversely, a loss of endogenous APP causes a decrease in spine density (Lee et al., 2010). Although the molecular mechanisms are not yet fully understood, the current knowledge clearly suggests an essential physiological function of trans-interacting full length APP in synapse organization.

Despite the well-documented essential functions of APP/APLPs at the synapse, there is little knowledge of the molecular signals activated by APP/APLPs either functioning as putative ligands or as cell surface-associated receptors. The identification of receptor(s) responsible for sAPP-dependent signaling may shed light on the molecular mechanism underlying sAPP function at the synapse. In contrast, studies of intracellular APP-binding proteins have already provided some interesting insights on the molecular mechanisms by which full-length APP may transmit synaptic signals. Here, we summarize current knowledge of the synaptic functions of APP-binding proteins. Protein-based studies used to identify APP interactors have yielded a long list of candidate proteins involved in many different pathways. Aside from a few interesting reports highlighting the putative interaction of APP with G-protein mediated signaling (Milosch et al., 2014; Ramaker et al., 2016), the proteins most commonly identified in these studies bind the YENPTY APP internalization motif. This review is a discussion of our knowledge of the synaptic role of YENPTY APP-binding proteins.

## APP/APLP Binding Proteins and Synaptic Function

Synapse formation and maintenance involves homo- and heterotypic interactions of Synaptic Cell Adhesion Molecules (SAM), including APP/APLP (Siddiqui and Craig, 2011), extracellular matrix components, extracellular ligands such as soluble APP fragments and other growth factors, as well as their adjacent receptors (Deyts et al., 2016). Herein, we present the signaling pathways involved in synapse formation, synaptic plasticity and synaptic neurotransmission in which APP-binding proteins participate, with a particular focus on the signaling events in which APP intracellular YENPTY domain binding proteins may play a role to alter synaptic function. This includes their role in well-established signal transduction pathways and their impact on cellular pathways, such as endocytosis, that are known to participate in signaling at the synapse (Fassio et al., 2016). The APP YENPTY domain binding proteins discussed include the X11/Munc-18-interacting proteins (Mints), FE65 proteins, Dab1, Numb/Numbl and Gulp1/CED-6, all capable of binding APP and other receptors through phosphotyrosine binding (PTB) domains (King et al., 2004; Wolfe and Guénette, 2007; Hao et al., 2011).

### Reelin Signaling

The large extracellular protein reelin is best known for its role in neuronal migration in the developing cortex. Reelin interaction with the lipoprotein receptors apolipoprotein E receptor 2 (ApoER2) and very low-density lipoprotein receptor (VLDLR) initiates a signaling cascade through tyrosine phosphorylation of bound Dab1, an adaptor protein that is essential for neuronal positioning in the developing mouse brain (D’Arcangelo et al., 1999; Hiesberger et al., 1999; Howell et al., 1999; Trommsdorff et al., 1999). Dendritic morphogenesis and excitatory synapse formation are also regulated by the reelin/ApoER2/VLDLR signaling pathway (Niu et al., 2004; Groc et al., 2007; Qiu and Weeber, 2007). In the adult brain, reelin signaling through ApoER2 alters the activity of postsynaptic glutamate receptors in hippocampal slices, affecting LTP and synaptic plasticity (Weeber et al., 2002; Beffert et al., 2005). These events are also dependent on tyrosine phosphorylation of the Dab1 adaptor protein and the subsequent recruitment of Src family kinases to phosphorylated Dab1, known as the canonical reelin signaling pathway (reviewed in Bock and May, 2016). In the adult hippocampus, Dab1 regulates synaptic plasticity (Trotter et al., 2013). The adult forebrain specific and excitatory neuron specific conditional Dab1 KO mice, used to demonstrate this role for Dab1, display deficits in associative (fear conditioning) and spatial learning, while demonstrating no other developmental abnormalities previously associated with loss of this protein (Trotter et al., 2013). However, spine area measurements of hippocampal CA1 apical dendrites were reduced in these conditional KO mice. Furthermore, impairments in both hippocampal LTP and reelin-induced LTP were observed and these were associated with deficits in the sustained activation of ERK2 following synaptic potentiation (Trotter et al., 2013). Thus, Dab1-mediated reelin signaling is important for synaptic plasticity.

Several lines of evidence support a functional interaction between APP and reelin signaling (Hoe et al., 2006, 2009; Pramatarova et al., 2008; Rice et al., 2013; Divekar et al., 2014). Despite, the identification of Dab1 as a cytosolic binding protein for APP (Homayouni et al., 1999), and evidence for a genetic interaction between APP and Dab1 (Pramatarova et al., 2008), there is no evidence supporting a role for APP binding to Dab1 in the transmission of an APP-dependent reelin signal. However, the increase in APP binding to ApoER2 and the post-synaptic density (PSD)-95 protein in primary cortical neurons treated with reelin suggests that APP may participate in reelin signaling as a co-receptor (Divekar et al., 2014). Given that ApoER2 association with itself is increased by reelin treatment and because receptor clustering is a known mechanism for activation of intracellular signaling cascades for other receptors such as EGFR, Trk and Ephrin, the reelin-dependent increase in APP binding to ApoER2 may play a role in reelin synaptic signaling (Divekar et al., 2014). Dab1 binding to both APP and ApoER2 may modulate downstream signals. In addition, FE65, which also binds the NPXY recognition motif in ApoER2, may compete with Dab1 in this cellular context, adding another level of complexity to the regulation of this signaling cascade (Hoe et al., 2006).

### Notch Signaling

The canonical Notch signaling pathway involves γ-secretase cleavage of Notch to produce the Notch intracellular domain (NICD) fragment, which is transcriptionally active. Notch signaling is regarded as a developmental signaling pathway for regulating stem cell maintenance and differentiation (Hori et al., 2013). It also plays a role in neurite outgrowth, dendritic arborization in immature neurons and synaptic plasticity in the adult brain (reviewed by Ables et al., 2011; Giniger, 2012). In mature pyramidal neurons, Notch signaling plays a role in regulating filopodia and spine densities (Dahlhaus et al., 2008; Alberi et al., 2011). Synaptic activity leads to an Arc-dependent increase in Notch and NICD levels (Alberi et al., 2011). Furthermore, downregulation of Notch in the hippocampus leads to impaired LTP and enhanced long-term depression (LTD; Wang Y. et al., 2004; Alberi et al., 2011), suggesting a role for Notch signaling in synaptic plasticity. Spatial learning deficits in the Morris Water Maze (MWM) and memory deficits in the Y-maze were also reported for mice in which Notch is knocked out in mature neurons (Alberi et al., 2011). Collectively, these data suggest that Notch signaling plays a role in hippocampal synaptic function.

Notch signaling is highly regulated, with the outcome being partly dependent on crosstalk with other signaling pathways and the type of cell receiving the Notch activation signal. One example of this crosstalk occurs between the Notch and reelin signaling pathways, with stabilization of NICD resulting from reelin-Dab1 signaling (Hashimoto-Torii et al., 2008). Moreover, NICD overexpression is able to rescue the neuronal migration phenotype of mice lacking reelin (Hashimoto-Torii et al., 2008). This seems to be due to the effect of Notch signaling on the morphology adopted by neural precursor cells to facilitate cellular migration. Whether crosstalk between Notch and reelin signaling plays a role in synaptic plasticity is presently unclear.

Evidence for interaction between Notch and APP signaling pathways also exists. Several studies have reported a physical interaction between APP and Notch (Fassa et al., 2005; Fischer et al., 2005; Oh et al., 2005, 2010; Chen et al., 2006). The YENPTY domain of Notch as well as APP interact with Numb and Numb-like (Numbl; Roncarati et al., 2002). Numb is an endocytic accessory protein that regulates clathrin-mediated endocytosis of its cargo proteins (reviewed in Yap and Winckler, 2015) and the absence of Numb and Numbl reduces Notch endocytosis producing higher levels of Notch and Notch signaling. Numb was identified in *Drosophila* as a Notch binding protein that regulates cell fate determination through inhibition of Notch signaling (Gulino et al., 2010). However, the consequence of altering Numb levels differs between vertebrates and *Drosophila*, as it’s absence in vertebrates produces morphogenesis defects rather than the predicted increase in neurogenesis resulting from increased Notch signaling (Kuo et al., 2006; Rasin et al., 2007). In the absence of Numb and Numbl, adherens junctions are lost in radial glial cells due to abnormal cadherin localization. This alters cell polarity, producing detachment and ectopic localization of radial glial cells in the developing cortex (Rasin et al., 2007). Thus, Numb mediated trafficking of N-cadherin in the endocytic pathway participates in the maintenance of adherens junctions. Numb, which is also expressed in the adult mammalian cortex, hippocampal pyramidal cell layer and cerebellum (Stump et al., 2002), may participate in the regulation of the endocytic trafficking of its cargo proteins at the synapse. In support of this possibility, Numb has recently been shown to participate in mGlu1 mediated LTD in Purkinje cells (Zhou et al., 2015).

Numb/Numbl binding to the APP intracellular domain, AICD, alters nuclear signaling by repressing Notch activity (Roncarati et al., 2002). Further evidence supporting crosstalk between APP and Notch signaling comes from promoter-reporter activation experiments showing that AICD in the presence of FE65 can trans-activate Hes-1, a Notch1 target gene, while NICD can trans-activate KAI-1, a putative AICD target gene, in HEK293 cells (Fischer et al., 2005). Interestingly, NICD trans-activation of the Hes-1 promoter can also be enhanced by FE65 expression (Fischer et al., 2005). However, opposing effects of APP on Notch signaling were reported for different cell types, indicating that the APP/Notch signaling crosstalk is context dependent (Oh et al., 2010). This may be due to cell-type dependent splicing of Numb, since alternatively spliced isoforms of Numb differentially affect APP internalization into the endocytic pathway (Kyriazis et al., 2008) and thus AICD generation. It may also be due to the cellular complement of adaptor proteins shared by Notch and APP, such as Numb and FE65, as competition of these adaptor proteins for APP or Notch/NICD may alter downstream signals. Furthermore, APP binding to Notch may modulate Notch signaling strength by preventing Notch receptor ligand interactions (Roncarati et al., 2002; Oh et al., 2005; Chen et al., 2006). Further studies are needed to assess whether crosstalk between Notch and APP signaling plays a role in synaptic structure and/or plasticity.

**Figure 1:**
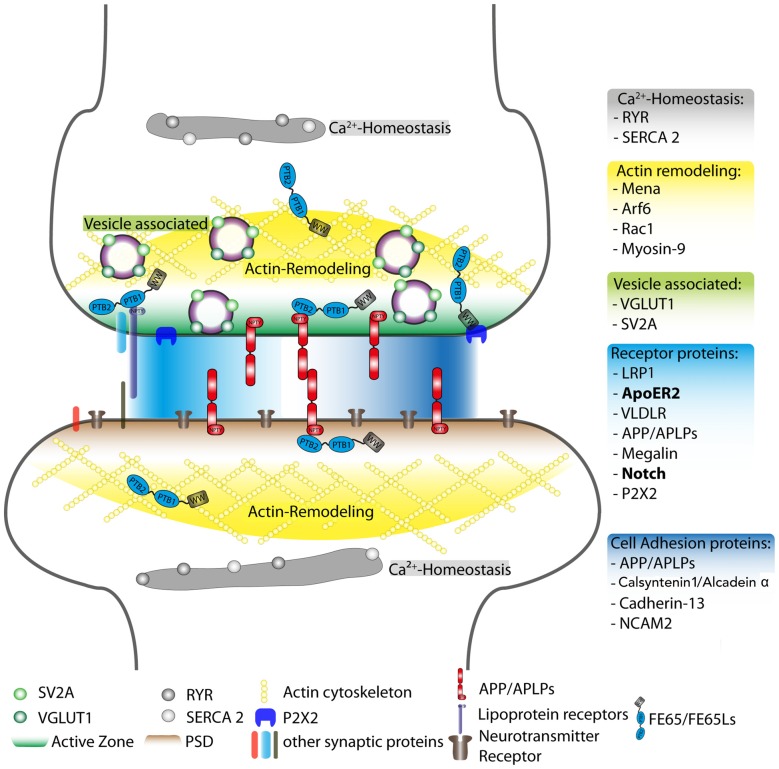
**Schematic overview of postulated FE65 protein family function at the synapse.** Reported interaction partners for the FE65 protein family are displayed at different synaptic sites, such as the active zone and the PSD. However, these interactions may also take place in other subsynaptic compartments. The individual FE65-interacting proteins were sorted into different functional units: regulation of Ca^2+^-homeostasis (gray), actin remodeling (yellow), vesicle-associated proteins involved in neurotransmitter release (green), cell adhesion (dark blue) and other surface receptor proteins (light blue). The FE65 binding receptors in bold are those implicated in signal transduction pathways known to alter synaptic function. The PSD, active zone, actin cytoskeleton and neurotransmitter receptors are highlighted in different colors. RYR, Ryanodine receptor; SERCA 2, sarcoplasmatic/endoplasmatic reticulum calcium ATPase 2; Arf6, ADP-ribosylation factor 6; Rac1, Ras-related C3 botulinum toxin substrate 1; VGLUT1, vesicular glutamate transporter 1; SV2A, synaptic vesicle glycoprotein 2A; LRP1, low-density lipoprotein receptor-related protein; ApoER2, apolipoprotein E receptor 2; VLDLR, very low-density lipoprotein receptor; APP, amyloid precursor protein; APLPs, APP-like proteins (1 and 2); P2X2, P2X purinergic receptor 2; NCAM2, neural cell adhesion molecule 2; L1CAM, neural cell adhesion molecule L1; PSD, post-synaptic density; PTB1 and PTB2, phosphotyrosine-binding domain 1 and 2; WW, protein domain containing two tryptophans.

The association of FE65 proteins with receptors such as ApoER2 and Notch/NICD is shown in Figure 1 as a possible mechanism by which FE65 may function at the synapse.

### Adhesion Proteins

Adhesion proteins that form complexes with APP, such as N-cadherin and calsyntenin/alcadeins, are implicated in synaptic contact formation and synaptic plasticity (Tang et al., 1998; Togashi et al., 2002; Arikkath and Reichardt, 2008; Pettem et al., 2013; Ster et al., 2014).

The classical cadherins participate in cell adhesion and communicate with their intracellular binding partners, the catenins, to link adhesion to intracellular pathways. The cadherin/catenin complex localizes to synapses where it regulates activity dependent spine remodeling (Arikkath and Reichardt, 2008; Bian et al., 2015). Although co-immunoprecipitation experiments demonstrate N-cadherin binding to APP (Asada-Utsugi et al., 2011), a role for N-cadherin/APP interactions in cadherin-modulated synaptic events has not been reported. However, N-cadherin binds the APP YENPTY binding protein, Numb, which plays a role in mGlu1 mediated LTD in Purkinje cells (Zhou et al., 2015). Thus, the ratio of APP-Numb and N-cadherin-Numb interactions may alter synaptic transmission.

In addition to classical adhesion molecules, there are a number of synaptic adhesion complexes that induce synaptic differentiation, a classic example is presynaptic neurexin binding to postsynaptic neuroligin. The cadherin related protein family member, Calsyntenin-3/Alcadein β, which is highly expressed in interneurons, forms a functional complex with α-neurexin that promotes calsyntenin-3 mediated presynaptic differentiation of inhibitory synapses (Pettem et al., 2013; Um et al., 2014). Calsyntenin-1 and -2 do not share this effect (Um et al., 2014). However, the observation that knockdown of all three calsyntenin proteins is necessary for decreased inhibitory synaptic transmission in both cultured hippocampal neurons and layer II/III somatosensory cortical neurons *in situ* suggests that all three family members redundantly regulate inhibitory synapse formation and function (Um et al., 2014).

Calsyntenin-1/Alcadein α forms a ternary complex with APP and the APP YENPTY binding protein, X11L. The formation of this ternary complex suppresses secretase cleavage of both APP (Araki et al., 2003) and Calsyntenin 1/Alcadein α (Araki et al., 2004). Furthermore, the γ-secretase cleavage product of Alcadein α, AlcαICD, competes with APP for FE65 binding and FE65 stabilizes AlcαIACD, similar to its stabilization of AICD (Kimberly et al., 2001; Araki et al., 2004). This competition may lead to regulation of AICD-mediated signaling. Although, the impact of Calsyntenin-1/Alcadein α cleavage on synaptic function is unknown its putative binding to FE65 at the synapse is highlighted in Figure 1.

Given that APP/APLP trans-dimerization is implicated in establishing synaptic contacts (Soba et al., 2005; Wang Z. et al., 2009; Prox et al., 2013; Klevanski et al., 2014), while factors that increase APP processing such as shedding (Stahl et al., 2014) or activity-dependent Aβ generation may be important for synaptic remodeling, a better understanding of the integration of Notch and reelin signaling on APP processing, signaling and metabolism at the synapse and the role of cross-talk between APP and other synaptic adhesion molecules seems warranted, as these may contribute to synaptic plasticity.

### Gulp1 and Endocytosis

Gulp1/CED-6, a YENPTY APP-binding protein, is a neuronal protein found in synaptosome-enriched fractions of rat brain, where it co-localizes with clathrin-coated vesicles (Martins-Silva et al., 2006). Gulp is involved in trafficking in the endocytic pathway enhancing APP processing and Aβ generation when overexpressed (Kiss et al., 2006; Hao et al., 2011). Furthermore, Gulp associates with and positively regulates ADP-ribosylation factor 6 (Arf6; Ma et al., 2007), a small GTPase that regulates clathrin and caveolin-independent endocytic trafficking of BACE1 in the somatodendritic compartment of neurons, where BACE1 encounters APP (Ma et al., 2007; Sannerud et al., 2011). Thus, Gulp/APP interactions might regulate synaptic levels of APP and its proteolytic products by regulating APP intracellular trafficking at the synapse.

### Regulation of APP Intracellular Complexes through Phosphorylation

APP-dependent modulation of synaptic structure and function may occur through alternative splicing of APP or phosphorylation of the APP C-terminus, thereby altering interaction of APP/APLP with their binding proteins (Kyriazis et al., 2008; Tamayev et al., 2009; Dunning et al., 2016). Alternative splicing of APP and its homologs is complex, but detailed investigations in the context of the putative physiological functions of APP are lacking (Pandey et al., 2016). The APP intracellular tail encompasses three Tyr and five Ser/Thr putative phosphorylation sites, of which two of the Tyr residues (Tyr682 and Tyr687) and three of the Ser/Thr sites (Thr654, 668 and Ser655) can exist in a phosphorylated state. These phosphorylation sites are docking sites for different adaptor proteins and at least for some of these, Tyr682 and Thr668, an influence on the binding of Shc and Grb2 or FE65, respectively, with full-length APP, and/or the α- and β-secretase derived APP C-terminal fragments has been documented (for review, see Schettini et al., 2010). The AICD interactome was also found to differ depending on phosphorylation of Tyr682 and Thr668 (Tamayev et al., 2009). Likely, the physiological relevance of the different sites can only be understood in specific signaling contexts and should include analysis of both APP phosphorylation and phosphorylation of their interacting proteins. More research will be required for a better understanding of these networks in the context of synapse formation and function.

The studies described above provide information on how APP-binding adaptor proteins contribute to signaling pathways implicated in synaptic function. Interestingly, X11 and FE65 proteins modulate signaling in these pathways. The remainder of this review focuses on the X11 and FE65 proteins and discusses their significance for synaptic function in light of recent KO studies. The synaptic phenotypes identified offer specific contexts in which to study the interplay between APP-binding PTB adaptor proteins and the additional ligands they bind on synaptic signaling.

## X11/Mint Mutant Mice

The APP-interacting proteins, X11, X11L and X11L2, bind to the YENPTY motif in the cytoplasmic region of APP (Borg et al., 1996; McLoughlin et al., 1999; Tanahashi and Tabira, 1999; Tomita et al., 1999). Interaction of X11 and X11L with Munc-18 (Munc-18-interacting protein (Mint)), a protein mediating membrane-vesicle fusion, was also reported (Okamoto and Südhof, 1997). Hence, multiple nomenclatures exist for this protein family: X11/X11α/Mint1, X11L/X11β/Mint2, and X11L2/X11γ/Mint3. In this review article, we refer to these proteins by their original nomenclature—X11, X11L and X11L2 (Duclos et al., 1993).

All three X11 proteins contain a conserved C-terminus, which consists of a phosphotyrosine-interaction/binding (PTB) and two PDZ (PSD95, *Drosophila* disc large tumor suppressor (Dlg1), and zonula occludens-1 protein (zo-1)) domains, mediating different types of protein–protein interactions. The X11 proteins diverge in the N-terminus, where X11 and X11L display an additional Munc-18 interacting domain and where only X11 bears a CASK-interacting domain (Okamoto and Südhof, 1997, 1998; Butz et al., 1998; Borg et al., 1999). Further, X11 and/or X11L associate with different interaction partners, including Kalirin-7 and XB51/NECAB3 (Lee et al., 2000; Jones et al., 2014). X11L is exclusively expressed in neurons, whereas X11 is found predominantly in the brain, but is also expressed in the pancreas, testis and paranephros (Motodate et al., 2016). Notably, some neurons, such as Purkinje cells showed only expression of X11, whereas X11L2 was found ubiquitously expressed, with substantial amounts in the brain (Motodate et al., 2016). The X11 family proteins regulate intracellular trafficking of APP as well as other NPXY motif containing transmembrane proteins (Araki et al., 2003; Saito et al., 2008, 2011; Gross et al., 2013; Sullivan et al., 2014) and affect APP processing, including generation of the Aβ peptide (Borg et al., 1998; Tanahashi and Tabira, 1999; Tomita et al., 1999; Shrivastava-Ranjan et al., 2008; Caster and Kahn, 2013). Interestingly, X11L2 and to a lesser extent X11L are distributed between the cytosolic and nuclear fractions, whereas X11 is recovered mostly in the cytosolic and membrane fractions. Thus, X11L2 might function as a transcriptional co-activator (Sumioka et al., 2008).

In a recent study, it was shown that all X11 family proteins are involved in activity dependent regulation of surface APP levels (Sullivan et al., 2014). Neuronal activity was associated with APP endocytosis followed by increased APP levels at the surface. This is highly interesting, as elevated APP cell surface levels were shown to increase APP synaptogenic activity (Stahl et al., 2014). In addition, X11 overexpression increases excitatory synaptic activity and activity dependent APP endocytic trafficking and Aβ generation (Sullivan et al., 2014). These data are consistent with the hypothesis, that X11/APP interactions may regulate activity-dependent synaptic remodeling.

X11 loss of function analyses revealed movement impairments and a decrease in GABAergic neurotransmission in KO mice (Ho et al., 2003, 2006). Further, X11-KO mice showed alterations in dopaminergic neurotransmission (Mori et al., 2002). X11L and X11L2 single KO mice revealed no obvious deficits, but X11L is functionally redundant for X11, as 80% of X11/X11L DKO mice die early after birth and the surviving mice exhibit increased growth and aggravated motor impairments (Ho et al., 2003, 2006; Sano et al., 2009). Furthermore, mouse X11/X11L mutants exhibited impairments in presynaptic neurotransmitter release, as indicated by lowered basal neurotransmission and reduced miniature excitatory post-synaptic current (mEPSC) frequency (Ho et al., 2006). As paired pulse facilitation was decreased and synaptic density was unchanged, these data can be explained by a decrease in synaptic vesicle release probability in X11/X11L DKO neurons (Ho et al., 2006). These data argue that the impaired synaptic vesicle release might be due to loss of interaction between X11/X11L and Munc-18. Consistently, the additional loss of X11L2, a family member lacking the Munc-18 binding site, did not aggravate the synaptic phenotype of X11/X11L DKO mice (Ho et al., 2006).

Interestingly, X11 single KO mice exhibit an increased paired-pulse depression at inhibitory synapses (Ho et al., 2003), consistent with an increased release probability, whereas analysis of X11/X11L/X11L2 KO neurons suggests a decreased release probability at excitatory synapses. This observation suggests that X11 may play a more specialized function at inhibitory synapses, whereas at excitatory synapses X11 and X11L might exhibit overlapping functions. Consistently, X11 is highly expressed in interneurons (Ho et al., 2003). However, other compensatory mechanisms may occur, for example, X11/X11L/X11L2-deleted neurons show increased levels of FE65, FE65L1 and FE65L2 proteins suggesting that X11 and FE65 proteins are functionally related (Ho et al., 2006). As X11 and FE65 proteins both contain a PTB domain, mediating binding to APP, it is conceivable that X11 and FE65 proteins are partially redundant for an APP-mediated function at the synapse (Ho et al., 2006). However, in a recent study no alterations in paired pulse facilitation were observed in FE65/FE65L1 DKO mice (Strecker et al., 2016). Alternatively, the functional overlap of X11 and FE65 may occur in dendritic spines. Levels of the AMPA-type glutamate receptor, GluR1, are increased in cortical neurons with acute deletion of X11 protein family members and the postsynaptic localization of the AMPA-type receptor GLR1 of *Caenorhabditis elegans* is impaired in Lin-10/X11 mutant interneurons (Rongo et al., 1998). Furthermore, X11 localizes to the mobile fraction of the PSD in excitatory cortical neurons where it interacts with Kalirin-7, a guanine-nucleotide exchange factor (GEF) that regulates Rac1 localization and function (Jones et al., 2014).

## FE65/FE65L1 Mutant Mice

The FE65 protein family, consisting in mammals of FE65, FE65-like 1 (FE65L1) and FE65-like 2 (FE65L2), are scaffolding/adaptor proteins able to form multi-molecular complexes that function in many cellular processes, such as calcium homeostasis (Nensa et al., 2014), actin remodeling and nuclear signaling (recently reviewed in Chow et al., 2015). All three FE65 proteins share conserved protein-protein interaction/binding motifs, namely the N-terminal WW-domain and the two C-terminal phosphotyrosine-binding domains 1 and 2 (PTB1, PTB2; Meiyappan et al., 2007; Radzimanowski et al., 2008a,b). The complexity of the FE65 protein family is further increased by the existence of several splice variants (p90FE65, p60FE65), polymorphisms within FE65 and cleavage products driven by proteases (p65FE65, which has an up to 40-fold higher affinity for APP than p97FE65; Hu et al., 1999, 2002, 2005; Domingues et al., 2011; Saeki et al., 2011; Golanska et al., 2013; Loosse et al., 2016). However, little is known about the specific localization and functions of these FE65/FE65L1/FE65L2 isoforms. Future experiments with specific antibodies against the different FE65 family members as well as their individual splice variants and processing products, might help clarify these questions.

FE65 and its family members interact with the intracellular domains of APP/APLPs (Fiore et al., 1995; Guénette et al., 1996; Duilio et al., 1998). As FE65 is predominantly expressed in the brain, similar to APP695, it has been studied more extensively than the more widely distributed FE65L1 and FE65L2 (Kesavapany et al., 2002; Guo et al., 2012). However, during mouse brain development FE65 expression clearly differs from APP. Whereas APP is upregulated during development until the first postnatal week, FE65 levels begin to decline after embryonic day 15 and increase again progressively from post-partum day 10 to adulthood (Sandbrink et al., 1997; Kesavapany et al., 2002). Interestingly, histological examination of FE65 or FE65L1 KO mouse brains revealed no abnormalities, while mice lacking both FE65 and FE65L1 resemble the APP/APLP1/APLP2 triple-KO (TKO) mouse phenotypes, exhibiting among other phenotypes, ectopic neurons and axonal pathfinding defects (Herms et al., 2004; Guénette et al., 2006). These data suggest that FE65 proteins mediate APP protein function in the developing brain possibly through transmission of an APP-dependent signal necessary for brain development. An alternative possibility is that loss of the FE65 proteins leads to APP-dependent sequestration of PTB-binding adaptor proteins essential for brain development.

The FE65 interaction with Mena/Vasp proteins, regulators of actin dynamics, is of interest because Mena KO mice have axonal pathfinding defects and improper positioning of neurons in the developing brain that bear resemblance to phenotypes observed in FE65/FE65L1 DKO and the APP/APLP1/APLP2 TKO mice (Lanier et al., 1999; Goh et al., 2002; Herms et al., 2004; Guénette et al., 2006). Recovery of a tripartite complex of FE65, Mena and APP and the co-localization of these proteins in growth cones and synapses suggest a neuronal function for this complex (Sabo et al., 2003; Ikin et al., 2007). Adenovirus-mediated expression of interaction-deficient FE65, bearing mutations that either abrogates PTB2 domain interactions (APP) or WW domain interactions (Mena/Vasp), altered axon branching (Ikin et al., 2007) suggesting a role for such complexes in neurite outgrowth. Functional analyses to determine whether APP-FE65-Mena/Vasp or FE65/Mena/Vasp complexes are present at the synapse would be a first step towards addressing a putative role for this complex in synaptic function.

Our recent detailed *in vivo* study examining FE65 protein family function using learning behavior analyses, immunohistological staining and electrophysiological measurements of different FE65/FE65L1 protein family KO mice provides further insights into the role of FE65 protein family members in the central and peripheral nervous system (CNS, PNS) that again show phenotypes similar to APP protein family KO mice (Strecker et al., 2016). Impairments in the maintenance of LTP in the Schaffer collateral pathway of FE65/FE65L1 DKO mice suggest that these proteins play a role in synaptic plasticity (Strecker et al., 2016). Although the FE65 single KO mice showed a trend towards decreased post-tetanic potentiation, maintenance of LTP was not significantly different from WT and no deficits were observed in FE65L1KO mice. A previous study of the isoform specific p97FE65 KO mice (lacking the longest FE65 isoform, p97, but simultaneously overexpressing six-times more of the shorter isoform, p60) reported early-phase LTP dysfunction (Wang Y. et al., 2009). Collectively these data support overlapping functions for FE65 and FE65L1 in synaptic neurotransmission. Interestingly, comparable potentiation rates have been observed in LTP measurements of acute hippocampal slices of APPΔCT15-DM mice (APP lacking the last 15 amino acids KI—APLP2 KO mice; Klevanski et al., 2015) pointing towards a shared function for FE65 and APP at the synapse. A role for FE65 proteins at the synapse is further supported by FE65 interaction with SV2, a synaptic vesicle protein, as well as sarcoplasmatic/endoplasmatic reticulum calcium ATPase (SERCA) and ryanodine receptor (RYR; Nensa et al., 2014), involved in calcium release/homeostasis in synapses under normal physiological conditions (reviewed in Mendoza-Torreblanca et al., 2013; Del Prete et al., 2014; Elaïb et al., 2016). Interestingly, dysregulation of calcium homeostasis is discussed in pathological conditions of AD (reviewed in Small, 2009), which may involve dysregulation of this aspect of FE65 protein function.

p97FE65 KO mice displayed deficits in cognitive behavior in non-spatial learning tasks and showed significant impairments in hidden platform and reversal learning in the MWM spatial learning test (Wang B. et al., 2004; Wang Y. et al., 2009). However, no memory deficits were observed for these mice. In contrast, memory deficits were observed in the MWM test for the FE65 KO (lacking both p60 and p97 isoforms) and FE65L1 KO mice in our study (Strecker et al., 2016). Confounding behaviors for locomotion analyses and possible loss of vision in FE65/FE65L1 DKO mice (Suh et al., 2015; Strecker et al., 2016) made it impossible to interpret the MWM spatial learning deficits observed for FE65/FE65L1 DKO mice.

Additional insights into the molecular mechanisms by which loss of the FE65 proteins results in the observed phenotypes comes from our knowledge of the function of their binding partners (Chow et al., 2015). The functions in which FE65 protein family members may participate include effects on actin cytoskeleton dynamics (Ermekova et al., 1997; Perkinton et al., 2004; Ward et al., 2010), Ca^2+^ homeostasis (Nensa et al., 2014), APP mediated signaling (discussed in more detail below), nuclear signaling via Tip60 (Cao and Südhof, 2001) and the response to DNA damage (Minopoli et al., 2012).

Early studies suggesting a role for FE65 in transcriptional regulation came from the identification of the histone acetyltransferase, Tip60 and the transcription factors, CP2/LSF/LBP1 and SET, as FE65 PTB1 domain binding proteins (Zambrano et al., 1998; Cao and Südhof, 2001; Telese et al., 2005). Despite intensive studies addressing AICD/FE65-regulated gene expression, there is a lack of consensus for most of the identified target genes (Hébert et al., 2006; Waldron et al., 2008). For a list of gene targets including those supported by promoter binding studies see Pardossi-Piquard and Checler (2012). These conflicting results may be due to the different experimental systems studied and the possibility that FE65 transcriptional regulation only occurs in specific physiological contexts. In support of this possibility, a recent study showed that FE65 is involved in epigenomic regulation of specific transcriptional programs implicated in the response to DNA damage (Ryu et al., 2015).

With respect to the role of FE65 proteins in synaptic function, the small GTPase, ARF6, which influences endocytic and membrane trafficking in neurons, is an intriguing FE65 interactor that may form an APP-FE65-Arf6 tripartite complex (Sannerud et al., 2011; Cheung et al., 2014; Tang et al., 2015). FE65 preferentially binds to ARF6 in its inactive GDP-bound form and stimulates the activation of ARF6 (Cheung et al., 2014). ARF6 is involved in synaptic function via regulation of AMPA receptor trafficking and synaptic plasticity during NMDA receptor-mediated LTP (Oku and Huganir, 2013). It also participates in NMDA-dependent LTD (Scholz et al., 2010) and regulates the cycling and readily releasable pool (RRP) of synaptic vesicles at the presynaptic site (Tagliatti et al., 2016). A recent study points towards a bi-directional function for ARF6 in spine formation and maintenance that is dependent on neuronal maturity and activity (Kim et al., 2015). In immature neurons expression of genes involved in cell motility and actin cytoskeleton organization are up-regulated by ARF6, while in mature neurons expression of genes important for neuronal activity such as synaptic transmission are up-regulated by ARF6 (Kim et al., 2015). Furthermore, synaptic activity reverses these effects indicating that ARF6 mediated signaling may play a role in synaptic plasticity (Kim et al., 2015). Interestingly, the interaction of FE65 and ARF6 influences ARF6 signaling to Rac1 (Cheung et al., 2014), which is implicated in neuronal outgrowth and spine structural plasticity (Cheung et al., 2014; Kim et al., 2015). In addition, Rac1 was previously reported to interact with FE65 and regulates its expression (Wang et al., 2011). Both Arf6 and Rac1 are included in Figure 1 as FE65 binding proteins that may contribute to FE65 function at the synapse. Knockdown of ARF6 also affects neuronal migration in the developing cortex (Hara et al., 2016). Thus, the FE65/ARF6 interaction and its effects on ARF6 signaling are consistent with many of the phenotypes observed in FE65/FE65L1 DKO and APP/APLP1/APLP2 TKO mouse brains. Further research in this direction should help determine the contribution of the FE65/ARF6 pathway to the phenotypic similarities observed between FE65/FE65L1 DKO and APP/APLP1/APLP2 TKO synaptic defects in the hippocampus.

FE65/FE65L1 KO and various APP KO mouse models share common impairments in NMJ formation with reduced pre- and postsynaptic areas and deficits in apposition of the pre- and postsynapse (Li et al., 2010; Weyer et al., 2011; Klevanski et al., 2014, 2015; Strecker et al., 2016). These are aggravated in FE65/FE65L1 DKO compared to FE65 or FE65L1 KO mice NMJs (Strecker et al., 2016), possibly leading to muscle degeneration/denervation (Suh et al., 2015) and the locomotion deficits and impairments in strength observed in these mice (Strecker et al., 2016).

APP interaction with low-density lipoprotein receptor-related protein 4 (LRP4), a component of the postsynaptic LRP4/MUSK/Agrin complex, is important for Acetylcholine-receptor patterning and stabilization at postsynaptic sites of the NMJ (Choi et al., 2013). Given that FE65 interaction with the intracellular domain of many lipoprotein receptors has been demonstrated (Gotthardt et al., 2000; Hoe et al., 2006; Alvira-Botero et al., 2010; Dumanis et al., 2012), the observation that the LRP4 ectodomain is sufficient for pre- and post-synaptic differentiation of the NMJ indicates that any contribution FE65 may have to this pathway may be via its interaction with APP in an APP/LRP4 tripartite complex (Gomez and Burden, 2011).

## Concluding Remarks

To gain insights into the molecular mechanisms by which APP functions at the synapse, we have re-examined the cytosolic APP interactome literature. Taking the different interaction partners into account, we highlighted some putative signaling pathways, involving Reelin, Notch and cell adhesion proteins, in which APP-interactors may participate to modulate synaptic function. PTB-containing interactors that bind the YENPTY motif in the APP-C terminus are the most prominently studied. Comparisons of several APP mutant mouse models that either lack or bear a mutation in the YENPTY motif, to X11 or FE65 KO mouse models reveal a surprisingly high degree of similarity between APP mutant mice and FE65 protein family KO mice (Table 1). Therefore, we conclude, that Fe65 family proteins play a pivotal role in APP function and have outlined possible cellular events in which APP-FE65 signaling may operate at the synapse (Figure 1). In future it will be important to evaluate those putative pathways and to investigate in more detail the regulation of APP-FE65 interactions at the synapse.

**Table 1:** **Phenotypic comparison of X11 and FE65 protein family knockout (KO) mice to amyloid precursor protein (APP) mutants lacking the X11/FE65 interaction domain**.

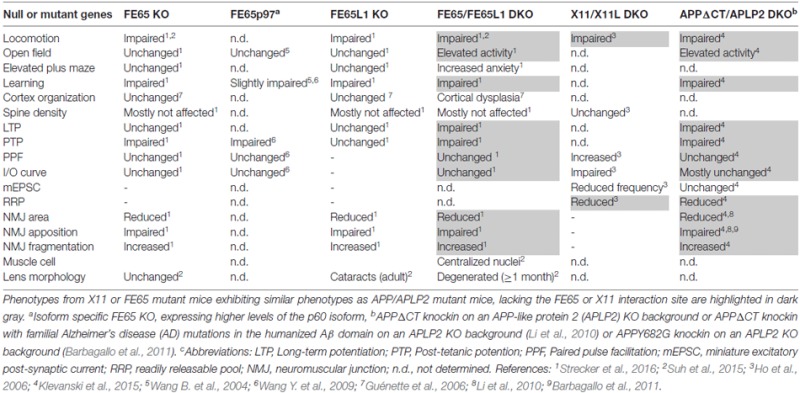

## Author Contributions

SG, PS and SK co-wrote the review.

## Conflict of Interest Statement

The authors declare that the research was conducted in the absence of any commercial or financial relationships that could be construed as a potential conflict of interest.
